# Supplementary material for: Association between coordinated counseling from both ASHA and Anganwadi Workers and maternal health outcomes: A cross-sectional study from Madhya Pradesh and Bihar, India
Source: PLOS Glob Public Health. 2024 Nov 13;4(11):e0003639. doi: 10.1371/journal.pgph.0003639 (PMC11560015; doi:10.1371/journal.pgph.0003639)
Supplement: S1 Table — (DOCX) [file pgph.0003639.s001.docx]

**Supplementary Material**

**Appendix: S1 Table**

S1 Table: Exposure and Outcomes Indicator Construction

|  | Outcome | Outcome Indicator | Outcome-specific counseling Indicator for ASHA, AWW and both |
| --- | --- | --- | --- |
| Antenatal period | Four or more antenatal care visits | 1 if the woman/mother had at least four ANC visits for index child, 0 otherwise | 1 if mother of child <12m received advice and counseling on health issues/danger signs during pregnancy; 0 otherwise |
|  | Birth preparedness | 1 if at least 75% of birth preparatory practices were followed by the woman /mother prior to the birth of indexchild, including identification of health facility for the delivery or in case of emergency during delivery, obtaining clean cloth for drying the baby, obtaining new blade to cut cord, saving money for delivery, arranging transport, etc. | 1 if mother of child <12m received advice on preparation for birth during pregnancy; 0 otherwise |
| Delivery | Institutional delivery | 1 if the woman/mother reported delivering the indexchild at a public/private facility, 0 otherwise | 1 if mother of child <12m received advice on facility delivery during pregnancy; 0 otherwise |
| Postnatal period | Postnatal care within 6 weeks of birth | 1 if woman/mother reported receiving two postnatal check-up at facility within 6 weeks of birth of indexchild, 0 otherwise | 1 if mother of child <12m received advice on health care and issues for mother and child in postpartum period; 0 otherwise |
|  | Contraceptive method | 1 if the woman/mother reported using any contraceptive method at the time of survey for purposes of preventing pregnancy, 0 otherwise | 1 if mother of child <12m received advice on family planning during pregnancy/ postpartum period; 0 otherwise |
| Contraceptive methods included the following: Female/male sterilization, intrauterine device (IUD), oral contraceptive pills, female / male condoms, injectable contraception, implants, diaphragm, foam/jelly or emergency contraceptive pills, standard days method, lactation amenorrhea, rhythm, withdrawal method.  Note:  ANC = Antenatal care  ASHA = Accredited Social Health Activist  AWW = Anganwadi Worker | | | |
