# Supplementary material for: Association between coordinated counseling from both ASHA and Anganwadi Workers and maternal health outcomes: A cross-sectional study from Madhya Pradesh and Bihar, India
Source: PLOS Glob Public Health. 2024 Nov 13;4(11):e0003639. doi: 10.1371/journal.pgph.0003639 (PMC11560015; doi:10.1371/journal.pgph.0003639)
Supplement: Attachment — Submitted filename: PGPH-D-24-01125_21_06_24.pdf [file pgph.0003639.s003.pdf]

PLOS Global Public Health

Association between coordinated counseling from both ASHA and Anganwadi Workers and maternal health outcomes in rural India: A cross-sectional study

--Manuscript Draft--

Manuscript Number:	PGPH-D-24-01125
Article Type:	Research Article
Full Title:	Association between coordinated counseling from both ASHA and Anganwadi Workers and maternal health outcomes in rural India: A cross-sectional study
Short Title:	Counseling from ASHA and Anganwadi Workers on maternal health
Corresponding Author:	Lakshmi Gopalakrishnan, PhD<br>University of California San Francisco<br>UNITED STATES
Order of Authors:	Lakshmi Gopalakrishnan, PhD
	Sumeet Patil, PhD
	Lia Fernald, PhD
	Dilys Walker, MD
	Nadia Diamond-Smith, PhD
Keywords:	convergence; integrated service delivery; community health workers; CHWs; South Asia; counseling; health service delivery
Abstract:	Objectives<br><br>Community Health Workers (CHWs), comprising various cadres such as Accredited Social Health Activists (ASHA) and Anganwadi Workers (AWW), within the framework of India's National Health Mission and Integrated Child Development Scheme, play pivotal roles in health promotion, counseling, and services rural areas. Previous research investigating the effectiveness of coordinated health promotion by different community health workers (CHWs) cadres on health practices is scarce. This study bridges this evidence gap by comparing the association between standalone and coordinated counseling by ASHA and AWW on a range of maternal health outcomes.<br><br>Methods<br><br>We use endline data from a previous impact evaluation conducted in 2019 consisting of 6635 mothers with children <12m from 841 villages in Madhya Pradesh and Bihar, India. We examine the association between outcome-specific counseling from either or both ASHA and AWW and maternal health practices such as four or more antenatal care (ANC) visits, birth preparedness, institutional delivery, postnatal care (PNC) visit, and contraceptive uptake using fixed effects logistic regression with robust standard errors corrected for multiple hypothesis tests.<br><br>Results<br><br>In our sample, 39.6% of women received four or more ANC visits, 31.2% adopted birth preparedness practices, 79.6% had institutional deliveries, 23.3% received postnatal care, and 19.5% adopted a postpartum contraceptive method. Receipt of outcome-specific coordinated counseling from both ASHA and AWW is associated with a greater prevalence of four outcomes (birth preparedness, institutional delivery, PNC visit, and contraception) compared to receipt of standalone counseling from either ASHA or AWWs.<br><br>Conclusion<br><br>We find evidence that health promotion by AWW is complementary to health promotion by the ASHA and collectively associated with improved health outcomes. This underscores the effectiveness of coordinated health promotion, which reinforces

Powered by Editorial Manager® and ProduXion Manager® from Aries Systems Corporation

	outcome-specific messaging from various CHWs and highlights the need for multisectoral and coordinated health promotion among different CHW cadres at the community level.<br><br>Trial registration number<br><br>https://doi.org/10.1186/ISRCTN83902145 ;<br><br>Date of registration<br><br>08/12/2016
**Additional Information:**	
**Question**	**Response**
**Financial Disclosure**<br><br>Enter a financial disclosure statement that describes the sources of funding for the work included in this submission and the role the funder(s) played. This includes grants and any commercial funding of the work or authors.<br><br>This statement will be typeset if the manuscript is accepted for publication.<br><br>*Please review the submission guidelines and the instructions link below for detailed requirements and guidance.*	This study is funded by Grant No. OPP1158231 from the Bill and Melinda Gates Foundation to the University of California, San Francisco and University of California, Berkeley. The funder (Bill and Melinda Gates Foundation) reviewed and approved the study design, but was not involved in data collection, data analysis, data interpretation, or writing of the report. The corresponding author had full access to all the data in the study and had final responsibility for the decision to submit for publication.
**Competing Interests**<br><br>On behalf of all authors, disclose any competing interests that could be perceived to bias this work.<br><br>This statement will be typeset if the manuscript is accepted for publication.<br><br>*Please review the instructions link below and PLOS Global Public Health's competing interests policy to determine what information must be disclosed at submission.*	Authors have no competing interests.
**Data Availability**	Dataset for this paper publicly available at Harvard Dataverse: https://dataverse.harvard.edu/dataset.xhtml?persistentId=doi:10.7910/DVN/XKCAIS

Before publication, Authors are required to make fully available and without restriction all data underlying their findings. Please see our PLOS Data Policy page for detailed information on this policy.<br><br>A **Data Availability Statement**, detailing where the data can be accessed, is required at first submission. Insert your Data Availability Statement in the box below.<br><br>Please see the data reporting section of our submission guidelines for instructions on what you need to include in your Data Availability Statement.<br><br>This statement will be typeset if the manuscript is accepted for publication.<br><br>*PLOS allows rare exemptions to address legal and ethical concerns. If you have legal or ethical restrictions, please use the box below to detail these in full sentences for the Journal team to consider.*	

Powered by Editorial Manager® and ProduXion Manager® from Aries Systems Corporation

Manuscript Click here to access/download;Manuscript;Manuscript_GH.docx

Association between coordinated counseling from both ASHA and Anganwadi Workers and maternal health outcomes in rural India: A cross-sectional study

Lakshmi Gopalakrishnan*, PhD, University of California, San Francisco, 550 16th Street, 3rd Floor San Francisco, CA 94158, USA
Sumeet Patil, PhD, NEERMAN, Unit 3, Mahendra Industrial Coop, Road 29, Sion East, Mumbai 400022, India
Lia Fernald, PhD, UC Berkeley School of Public Health, 2121 Berkeley Way, Berkeley, CA 94704, USA
Dilys Walker, MD, University of California, San Francisco, 550 16th Street, 3rd Floor San Francisco, CA 94158, USA
Nadia Diamond-Smith, PhD, University of California, San Francisco, 550 16th Street, 3rd Floor San Francisco, CA 94158, USA

*Corresponding author:
Lakshmi Gopalakrishnan
Lakshmi.gopalakrishnan2@ucsf.edu

Key words: convergence, integrated service delivery, community health workers, CHWs, South Asia, counseling, health service delivery

Abstract

Objectives: Community Health Workers (CHWs), comprising various cadres such as Accredited Social Health Activists (ASHA) and Anganwadi Workers (AWW), within the framework of India's National Health Mission and Integrated Child Development Scheme, play pivotal roles in health promotion, counseling, and services rural areas. Previous research investigating the effectiveness of coordinated health promotion by different community health workers (CHWs) cadres on health practices is scarce. This study bridges this evidence gap by comparing the association between standalone and coordinated counseling by ASHA and AWW on a range of maternal health outcomes.

Methods: We use endline data from a previous impact evaluation conducted in 2019 consisting of 6635 mothers with children <12m from 841 villages in Madhya Pradesh and Bihar, India. We examine the association between outcome-specific counseling from either or both ASHA and AWW and maternal health practices such as four or more antenatal care (ANC) visits, birth preparedness, institutional delivery, postnatal care (PNC) visit, and contraceptive uptake using fixed effects logistic regression with robust standard errors corrected for multiple hypothesis tests.

Results: In our sample, 39.6% of women received four or more ANC visits, 31.2% adopted birth preparedness practices, 79.6% had institutional deliveries, 23.3% received postnatal care, and 19.5% adopted a postpartum contraceptive method. Receipt of outcome-specific coordinated counseling from both ASHA and AWW is associated with a greater prevalence of four outcomes (birth preparedness, institutional delivery, PNC visit, and contraception) compared to receipt of standalone counseling from either ASHA or AWWs.

Conclusion: We find evidence that health promotion by AWW is complementary to health promotion by the ASHA and collectively associated with improved health outcomes. This underscores the effectiveness of coordinated health promotion, which reinforces outcome-specific messaging from various CHWs and highlights the need for multisectoral and coordinated health promotion among different CHW cadres at the community level.

Trial registration number: https://doi.org/10.1186/ISRCTN83902145;

Date of registration: 08/12/2016

Introduction

Despite significant progress, maternal mortality and neonatal mortality remain stubbornly high in India, at 99 per 100,000 and 23.7 per 1000 live births [1–3].For every one woman who dies from maternal causes, about 20 women experience morbidities such as postpartum depression, high blood pressure, and infections [4]. However, a significant proportion of these maternal, newborn mortality and morbidity can be prevented with improved care in the first 1000 days of life through increased coverage and quality of life-saving interventions such as antenatal care visits, folic acid supplementation, early initiation of breast feeding, exclusive breastfeeding, among others [5, 6]. There is sufficient evidence that community-based delivery platforms connected to local health facilities can expand coverage of essential interventions as well reduce inequities [5].

In many low-and-middle-income countries (LMICs), including India, community health workers (CHWs), operate at the frontlines in improving access to and increasing utilization of essential maternal, newborn and child health interventions through health promotion activities and counseling, acting as a link between community members and health systems [7–9]. Strengthening CHW programs remains an important strategy in achieving universal coverage of key community-based evidence–based interventions and health services by the year 2030 [7]. Prior systematic reviews have found that community mobilization, education, and home-based counseling by CHWs help improve maternal, neonatal and child health outcomes [7, 10, 11].

India has two flagship national programs with combined workforce of more than 2 million female CHWs maternal health and nutrition promotion and allied services. Accredited Social Health Activists (ASHAs) work under the National Health Mission (NHM) of the Ministry of Health and Family Welfare (MoHFW) and Anganwadi Workers (AWWs) are under the Integrated Child Development Services (ICDS) of the Ministry of Woman and Child Development (MWCD). Table 1 outlines the overlapping and distinct roles and responsibilities of ASHAs and AWWs.

Table 1: Roles and responsibilities of ASHAs and AWWs to promote the health of mothers and children [1,2]

	Accredited Social Health Activist	Anganwadi Workers
Ministry	Ministry of Health and Family Welfare (MoHFW)	Ministry of Woman and Child Development (MWCD)
Program	National Health Mission	Integrated Child Development Services
Scale	1.4 million	980,000+
Education	Completed at least 10 years of schooling	Completed at least 10 years of schooling
Population served	Provide services to pregnant and lactating women, adolescent girls and men at the village level, roughly catering to the population of ~1,000 in rural areas.	Provide services to pregnant and lactating women and children 0-6 years, adolescent girls via Anganwadi centers catering population of ~1000.
Overlapping responsibilities	- Conduct home visits to promote birth preparedness, institutional delivery, family planning, immunization, regular health check-up, nutrition for mother and child, and breastfeeding.<br>- Record keeping of basic details, services, health and nutrition status, immunizations, etc. on a common ICDS-NHM mother-child protection (MCP) card<br>- Mobilize women and children for monthly Village Health and Nutrition Day and support service delivery (e.g., immunization, health check-up, distribution of iron folic acid, calcium tablets, etc. done by the Auxiliary Nurse and Midwife [ANM]) along with AWWs<br>- Promotion of incentives linked government schemes and benefits<br>- Promotion of water-sanitation-hygiene	

	Accredited Social Health Activist	Anganwadi Workers
	- Conduct home visits to promote nutrition and health for mother and child, pregnancy care, institutional delivery, immunization, exclusive breastfeeding, complementary feeding, diet diversity and adequacy.<br>- Record keeping of basic details, services, health and nutrition status, immunizations, etc. on a common ICDS-NHM mother-child protection (MCP) card<br>- Mobilize women and children for Plan for monthly Village Health and Nutrition Day and support service delivery along (e.g., immunization, health check-up, distribution of IFA/CA, etc. done by the ANM) with ASHAs<br>- Promotion of incentives linked government schemes and benefits<br>- Promotion of water-sanitation-hygiene	
Non-overlapping responsibilities	- Accompany pregnant women and children to healthcare facilities for check-up, delivery<br>- promote and accompany women and men for family planning procedures at healthcare facilities<br>- Provide DOTS for patients with TB.<br>- Carry essential provisions (ORS packets, TB medicines, iron and folic tablets, chloroquine<br>disposable delivery kits, oral contraceptive pills, condoms, sanitary napkins)<br>- Support ANMs in postnatal care check-ups, including homebased newborn care<br>- Universal screening, prevention, and management of non-communicable diseases	- Monthly growth monitoring of children <6 years (height/length, weight, arm circumference)<br>- Referral to ASHA / ANM for health complications, referral of severely malnourished children to nutrition rehabilitation centers.<br>- Community based management of malnourished children<br>- Provide early childhood training to children <3 years and non-formal pre-school education to 3–6 year-old children<br>- Provide monthly take home rations or food supplements to pregnant women, lactating mothers, and children<br>- Provide hot cooked meals at Anganwadi Center to pregnant women and children 6m-6 years<br>-Organize nutrition specific community events such as annaprashan (first solid meal fed to infants), Godbharai (baby shower), and invite ASHA to participate<br>- Carry ORS packets
Payment structure	Voluntary worker who receives performance-based incentives based on tasks completed. For instance, ASHA is paid USD 3.75 (INR 300) for each institutional delivery in rural areas.	Part-time contract worker who receives fixed monthly honorarium which varies by states, e.g., USD 55 (INR 4500) in Bihar, and USD 160 (INR 13000 in Madhya Pradesh)

[1] Compiled from various sources [12–18]
[2] Additional responsibilities, training, and payment amounts of the ASHAs and AWWs may vary by state.

Several studies from India have studied the role of CHWs in improving health practices and outcomes, but not the coordinated counseling or health promotion activities by different cadres of CHWs together. For example, few studies have identified positive impact of ASHA engagement or contacts in improving maternal health outcomes [13, 19–23]. Studies documenting AWW effectiveness have primarily relied on evaluation of ICDS program. A recent analysis of ICDS led by AWW highlights an improvement in usage of ICDS services between 2006 and 2016, however, the program did not successfully reach households from the poorest backgrounds and women with low schooling levels especially in states with the highest burden of undernutrition [24]. Further, the national survey data also suggests deficiencies in the ICDS system—only 67% of mothers reported receiving food supplements, growth monitoring, and pre-school education services from AWWs for children below six with wide variation between states [25].

Evidence on coordinated health promotion and integrated service delivery of CHWs between the ICDS and NHM program is scarce. A few studies that have controlled for the presence of other cadre of CHWs did not measure additive effects of receiving counseling from ASHA and AWW together. Rammohan and colleagues examined maternal engagement with any type of CHW cadres (ANM,ASHA,AWW, multipurpose worker) but did not delve into the separate impact of exposure to each of these CHWs [26]. Another study focused on the independent association of antenatal care check-ups provided by ASHAs, AWWs, and any other CHW on birthweight and mortality but did not examine additive association of receiving ANC from all CHWs [23]. Yet another study that examined the association between multiple types of health influences such as ASHA, AWW, ANM, family members, rural practitioners, among others and found that ASHAs were most effective in uptake recommended maternal health behaviors [27]. Only one qualitative study was identified, which explored the role of convergence between ASHA and AWW and underscored the significance of integrated service delivery as a catalyst for increased community participation and accountability [28]. there is a dearth of quantitative research that has systematically examined the effectiveness of coordinated health promotion initiatives led by different types of CHWs in improving maternal and newborn health outcomes.

The lack of evidence regarding coordinated service delivery and health promotion is concerning, particularly in light of the recognition of multisectoral collaboration or convergence of health and nutrition services as an essential strategy for achieving the global goals such as Universal Health Coverage and Sustainable Development Goals [29, 30]. There is growing recognition that the current challenges and inequities experienced by women and children will likely continue to be worsened by the impact of COVID-19, necessitating the imperative for multisectoral collaboration [31]. Moreover, this holds significant importance as the Ministries of Women and Child Development (MWCD) and Health and Family Welfare (MoHFW) have recently acknowledged the importance of collaboration between ASHA and AWW, along with the adoption of a unified behavior change communication strategy. This strategy encompasses joint planning and coordination in areas where the ASHA and AWW responsibilities intersect, with aim of improving maternal and health outcomes [32].

Our study seeks to bridge this evidence gap on the role of coordinated health promotion in context of counseling from both ASHAs and AWWs on maternal health outcomes in India. We hypothesize that coordinated counseling from both ASHA and AWWs will be associated with better health practices, compared to standalone counseling from either ASHAs or AWWs on four or more antenatal check-ups, birth preparedness, seeking postnatal care, adopting postpartum contraceptive method.

Data and Methods

Data

This uses secondary data from an endline survey conducted December 2018 through August 2019 across 12 districts of two states of northern India -- Madhya Pradesh (MP) and Bihar – to evaluate effectiveness of an mHealth intervention to digitally enable AWWs. More information is available in the published protocol [33] and the final impact evaluation paper [34]. The analytical sample consisted of 6,635 mothers with children <12 months from 852 villages from 12 districts (6 of which had implemented and 6 districts that had not implemented the mHealth

intervention) using propensity-score matched design. In each village, the list of beneficiaries available with the AWWs was used as a unit-level sample frame to randomly sample up to eight mothers of children <12 months. All study participants provided verbal audio-recorded informed consent before data collection. Trained female enumerators surveyed respondents using structured computer-assisted personal interviews. Through the research, the study team only had access to de-identified data.

Ethical approvals

Study protocols were reviewed and approved by institutional review boards at the University of California, Berkeley (Ref. No. 2016-08-9092), and the India-based Suraksha Independent Ethics Committee (Protocol No. 2016-08-9092). The trial is registered at https://doi.org/10.1186/ISRCTN83902145.

Measures

Exposures of Interest: Our main exposures were receipt of *outcome-specific standalone counseling* from ASHAs or AWWs reported by the surveyed mothers during their pregnancy, delivery, and postpartum periods with the recent child. A mother was considered to have received *outcome-specific coordinated counseling* if she reported receiving advice from both ASHA and AWW. Mothers were asked whether and from whom they received any counseling or advice on specific topics such as advice on issues/danger signs and check-up during pregnancy, advice on birth preparedness, advice on institutional delivery, advice on postnatal care within first 6 weeks of delivery, and advice on family planning.

Outcomes of interest: The outcomes of interest were whether the mother: (i) received four or more ANC check-ups, (ii) adopted birth preparedness practices (identification of facility, clean cloth for drying, saving money for delivery, etc.), (iii) delivered in a health facility, (iv) received at least two postnatal check-ups within six weeks of the delivery, and (v) adopted a contraceptive method post childbirth. Exact indicator construction of both exposure and outcome is listed in the Appendix (S Table-1) as supplementary material.

Covariates: We control for a several potentially confounding variables as informed by prior literature [13, 21]. Specifically, we control for the following socio-demographic variables in our models: women's age at time of survey (continuous), years of education (continuous), women's work outside the home (yes/no), total number of pregnancies (continuous), and woman's caste. For caste, following the Government of India classifications, we categorized scheduled tribe/scheduled caste as marginalized caste with other backward classes and general caste serving as the reference group. We control for wealth index (in quartiles) from the principal components analysis of household assets. We include fixed effects for the sub-district level to control for any effect of the mHealth intervention. This is important given that the mobile technology intervention was delivered at the district level as well as the sub-district level factors that may potentially affect both service delivery of CHWs and social environment that drives women's health decision making.

Analytical approach

We describe sample characteristics in terms of frequencies, proportions and/or median and interquartile range (IQR) of the exposure and outcome indicators and covariates discussed previously. For each outcome, we fit a fixed-effects logistic regression adjusted for covariates which could potentially bias the estimated association between the outcome and counseling from CHWs. Since the mHealth intervention was assigned at the district level, the fixed effect at sub-district level ensures that the associations are estimated in the original treatment and controlled sub-districts separately and then averaged over.

We quantify the marginal effects (or prevalence differences) and 95% Confidence Intervals (CIs) for each outcome using the *margins* post estimation command in Stata, which uses the delta method to estimate the standard errors of the marginal effect [35]. To test the one-sided hypothesis that effect of coordinated counseling from both AWW and ASHA is larger than that of counseling from either ASHA or AWW, we use one-sided Chi-square tests by using the post estimation *test* in Stata.

All regression models and post-estimation tests used clustered robust standard errors at the village level. Because we assessed association between coordinated counseling for five outcomes separately, we adjusted the p-values for multiple comparisons using Bonferroni correction [36]. We used $p < 0.05$ as statistical significance for all models. All analyses were conducted using Stata V.15 [37].

Results

Table 2 presents the descriptive characteristics of our study population. On average, women were young with 50% of the sample being below 24 years of age with majority of the population falling between 21 and 26. Despite the young age, the median number of pregnancies was 2, indicating that half of the women in the population have experienced fewer than 2 pregnancies. The level of the educational attainment was low with 50% having fewer than five years of schooling. Almost half (44.1%) of the sample were affiliated with the scheduled caste or tribe. Moreover, a mere 18% of the women reported engaging in paid work outside their households at the time of the survey. The sample was even split between the two states.

Table 2: Socio-demographics of the study population of women with a child 0-12 months (N=6635)

	**n**	**Value**
Age (median, IQR)	6,635	24 (21-26)
Gravida or number of pregnancies (median, IQR)	6,635	2 (1-3)
Education in years (median, IQR)	6,635	5 (0-9)
**Children age groups**		
0-3 months	1,533	23.0%
3-6 months	1,957	29.5%
6-9 months	1,719	25.9%
9-12 months	1,426	21.5%
**Engaged in paid work**	1,200	18.1%
**Caste**		
Scheduled Caste or Tribe	2,926	44.1%
Other Backward Class/General Caste	3,709	55.9%
**Wealth quartile**		

	n	Value
Quartile 1	1,682	25.4%
Quartile 2	1,655	24.9%
Quartile 3	1,652	24.9%
Quartile 4	1,646	24.8%
**State**		
Bihar	3,417	51.5%
Madhya Pradesh	3,218	48.5%

Figure 1 presents the proportion of mothers who received outcome-specific standalone counseling from ASHA, AWW or both (coordinated counseling). The most recalled topics of standalone counseling from ASHA and AWW were similar but with higher recall for ASHA—institutional delivery (from ASHA: 66.0%; from AWW: 48.0%), family planning (from ASHA: 66.0%; from AWW: 48.0%) and birth preparedness (from ASHA: 54.4%; from AWW: 46.2%). Coordinated counseling from both ASHAs and AWWs followed a similar pattern—institutional delivery (38.1%), family planning (38.1%), and birth preparedness (35.8%) topics were most recalled by the mothers.

Figure 1: Coverage of outcome-specific counseling from ASHAs and Anganwadi Workers (AWWs) in Bihar and Madhya Pradesh, N =6635

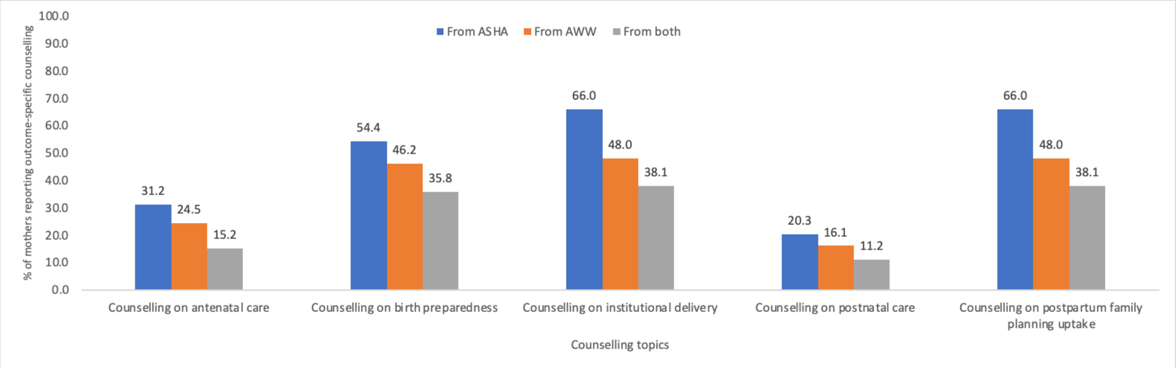

Table 3 presents the results from the main analyses to find association between receipt of outcome-specific counseling from CHWs and the health outcomes. Column (A) presents the mean of the outcomes for *the reference group* of mothers who did not receive any counseling on the given topic from the ASHA or AWW. Columns (B), (C), and (D) present increase in the proportion of mothers reporting an outcome compared to the reference group associated with receipt of the outcome-specific counseling from only the AWW, only the ASHA, or from both.

Contrary to our hypothesis, coordinated counseling on counseling on health issues/danger signs during pregnancy is not statistically significantly associated (-1.3 pp [95%CI: -4.2 – 1.4 pp]) with mothers receiving four or more ANC check-ups compared to the reference group mean of 36.8 percent. Standalone counseling from only ASHA or only AWW are also not associated with mothers receiving four or more ANC.

The results are largely as expected in terms of the other four outcomes. The proportion of mothers practicing birth preparedness is substantially higher among those who receive

coordinated counseling from both ASHA and AWW (20.5pp [95% CI: 17.3 – 23.7 pp] compared to reference group mean of 27.2 percent). The proportion of mothers practicing birth preparedness is also higher for standalone messaging from either ASHA (16.7 pp [95% CI: 13.5 – 20.0 pp]) or AWW (9.7 pp [95% CI: 5.1 – 14.1 pp]). The coordinated counseling is also more effective than standalone counseling from ASHAs and from AWWs.

In case of institutional delivery, the coordinated counseling is more effective than standalone counseling by AWW, but not more effective than that from ASHA. The association between coordinated counseling and institutional delivery is higher (8.7 pp [95%CI: 6.1 – 11.2 pp] compared to reference group mean of 76.4 percent) than standalone counseling from AWW (3.3 pp [95%CI: 0.2 – 6.3]).

In terms of postnatal care outcome, the association between coordinated counseling and postnatal care check-up within six weeks is also substantially greater (29.2pp [95% CI: 24.8 – 33.5 pp] compared to reference group mean of 19.6 percent) than standalone counseling from either ASHA (22.2 pp [95% CI: 18.4 – 26.1 pp]) or AWW (19.6 pp [95% CI: 14.6 – 24.5 pp]).

In the case of outcome on postpartum contraception use, the coordinated counseling is more effective than standalone counseling by AWW but not more effective than that from the ASHA. Further, both coordinated counseling and standalone counseling from ASHA were equally associated an increase in the proportion of mothers using a postpartum contraceptive method of 11.5 pp [95%CI: 8.7 – 14.4] and higher than the association of postpartum contraceptive use with standalone counseling from only AWW (7.7 pp [95%CI: 3.8 – 11.6 pp).

Table 3: Association between behavior-specific advice from ASHA and Anganwadi Worker (AWW) on maternal, newborn and child health outcomes using logistic regression models† (N=6635)

	**Mean in the reference group (mothers reporting no counseling from ASHA or AWW)**<br>**(A)**	**Received outcome-specific standalone counseling from ASHA**<br>**(B)**	**Received outcome-specific standalone counseling from AWW**<br>**(C)**	**Received outcome-specific coordinated counseling from both ASHA and AWW**<br>**(D)**	**Comparison of additive effect of coordinated counseling from both with standalone counseling from either ASHA or AWW**<br>**(E)**	
**Health outcomes reported by mothers**		*Prevalence Difference (B-A) (95% CI)*	*Prevalence Difference (C-A) (95% CI)*	*Prevalence Difference (D-A) (95% CI)*	*H0: D ≤ B*<br>*H: D > B*<br>*Chi² statistics*	*H0: D ≤ C*<br>*H: D > C*<br>*Chi² statistics*
Four ANC visits	0.368	0.009<br>(-0.024- 0.042)	- 0.030*<br>(-0.017 - 0.058)	-0.013<br>(-0.042- 0.014)	1.47	0.67
Birth preparedness	0.272	0.167***<br>(0.135 - 0.200)	0.097***<br>(0.051 - 0.141)	0.205***<br>(0.172 - 0.238)	5.50**	26.53***
Institutional delivery	0.764	0.068***<br>(0.044 - 0.093)	0.033**<br>(0.002 - 0.063)	0.087***<br>(0.061- 0.112)	1.5	9.83***
Postnatal care	0.196	0.222***<br>(0.184 – 0.261)	0.196***<br>(0.146 - 0.245)	0.292***<br>(0.248 - 0.335)	13.59***	29.80***
Contraceptive method	0.168	0.115***<br>(0.087 - 0.144)	0.0770***<br>(0.038 - 0.116)	0.115***<br>(0.085 - 0.145)	0.00	5.20*

*** p<0.01, ** p<0.05, *p<0.1 adjusted for multiple comparisons using Bonferroni correction; Robust 95%CI in parentheses
†Model adjusted for the following variables: maternal education, maternal age, caste, gravida, household wealth quintile, block-level characteristics.

Discussion

Overall, our findings indicate that receipt of coordinated counseling from both ASHA and AWW were associated with better uptake of maternal health outcomes. These results were robust to statistical tests considering Bonferroni corrections for multiple comparison for four out of five outcomes—birth preparedness, institutional delivery, postnatal care check-up and contraceptive uptake.

Our findings suggest that the magnitude of the association between standalone counseling from ASHAs and outcomes were higher than that for the standalone counseling from AWWs. Indeed, all the health outcomes included in this study were core to the NHM and thus led by ASHA, whereas for AWWs, the ICDS nutrition and child growth are the primary outcomes and maternal health outcomes are secondary [38, 39]. ASHAs also get incentives for mobilizing the community for the VHNDs, when pregnant women initiate ANC, for institutional delivery, postpartum home visits, and for promoting family planning methods unlike fixed renumeration for the AWW which can also explain why health promotion by ASHAs has stronger association with health practices [15].

Above findings are consistent with a recent study from rural Bihar that examined the role of multiple health influencers including ASHA, AWW, ANM, family, among others, and found that ASHAs consistently had the strongest association on mothers engaging with recommended health behaviors such as institutional delivery, early antenatal care registration, feeding colostrum and other postpartum behaviors [27] and prior studies documenting the effectiveness of ASHAs [13, 40]. ASHAs remain the primary contact for addressing the healthcare needs of women and children, offering guidance on topics such as birth preparedness, the significance of safe childbirth, and contraception [41]. Further, the performance-linked monetary incentives for ASHAs could be a driving factor in better quality of counseling [15], though we did not have measures for quality of counseling. A review that examined the role of CHWs who provide maternal health services found that that counseling from ASHAs were sought by the beneficiaries as they were more accessible and trusted with community members viewing them as “familiar faces” [42].

Regarding birth preparedness and postnatal care outcomes, our findings underscore the greater effectiveness of coordinated counseling when compared to standalone counseling provided by ASHA or AWW. Specifically ASHAs do not receive incentives for activities associated with birth preparedness practices. Their incentives are tied to promoting institutional delivery, encouraging antenatal care visits during pregnancy, and conducting postnatal care home visits, but not specifically for birth preparedness. Similarly, ASHAs receive incentives for conducting six postpartum home visits, which is distinct from the postnatal checkup at a health facility within the first six weeks. Our study suggests that when ASHA's incentives are not directly linked to a specific health, it appears that coordinated counseling with AWW is more effective. On the other hand, for outcomes such as institutional delivery and contraceptive uptake, where both ASHAs and beneficiaries both receive monetary incentives from the government, standalone counseling provided by ASHA is, statistically, as effective as the coordinated counseling.

In our study, we did not observe a significant association between counseling on check-up during pregnancy and four or more ANC visits. A national-level analysis of the ASHA program had found that while ASHAs were effective in helping women initiate their maternity care through any ANC, ASHAs were less effective in ensuring completion of services along the continuum of care such as four or more ANC visits [43]. A modeling study on government payments to ASHAs and actual compensation of ASHAs found that ASHAs were paid for even partial completion of ANC visits, highlighting the importance of correctly aligning incentives in motivating ASHAs to achieve the sequence of desired outcomes, such as four or more ANC visits [44]. Further, prior research has noted many social, cultural economic determinants such as lower maternal education, poor socio-economic status, poorer women's autonomy, and limited spousal support may also disrupt continuity in care, limiting the use of four or more ANC visits [45, 46]. Supply side factors such as low quality or a negative experience with a previous ANC visit may also reduce coverage of later ANC visits [47, 48]. Therefore, even though ASHAs receive incentives when pregnant women initiate ANC, their counseling may not ensure coverage of four or more ANCs and may not be able to overcome the structural barriers.

To our knowledge, this study is among the first to examine the standalone as well as coordinated counseling from multiple cadres of CHWs on maternal health outcomes. Most of the prior studies from India have focused only on either ASHA or AWW counseling and by failing to account for receipt of health and nutrition counseling from the other CHW may have overestimated the effectiveness of a single type of CHW [13, 20, 21, 40]. Some studies have controlled for engagement with other type of CHW [23, 26, 27, 49] but none studied the effect of coordinated health promotion despite their overlapping roles at the community-level.

Limitations and strengths

Our study has some limitations that most cross-sectional studies face but also some strengths compared to previous evidence on this topic. Our measurements relied on self-reported data by mothers and thus subject to recall bias and social desirability bias. Because the sample for this study is based on propensity score matching and a part of the sample was exposed to an mHealth intervention, the findings are strictly *not* representative of general population of mothers with children <12 months in Bihar and MP, but still based on a large sample of more than 6500 mothers from 841 villages. We draw upon a large and uniquely structured data that encompass a set of outcomes and outcome-specific counseling delivered by ASHAs and AWWs for pregnancy care, childbirth, and postnatal care, thereby enriching the depth and scope of our investigation rather than generic exposure or number of home visits by CHW as have been done in prior studies. The findings of this study are at best associations although we have attempted to control the selection bias to the extent possible with the available data. Our analyses also exclude health promotion by the ANMs because they primarily focus on provision of services such as health check-up, referrals, and immunizations and have responsibility of a population of 10000+ from multiple villages to give focused attention to health promotion unlike ASHAs and AWWs who operate at the village-level [50].

Conclusion

Our study adds to the existing literature about how health promotion by CHWs can change maternal health practices. In context of India, two national-scale programmes (NHM and ICDS)

are coordinating their activities at the community level through their respective CHWs. The coordinated counseling is strongly associated with better health practices except when the practices are depending on external factors and counseling alone is unable to overcome the structural barriers as was the case for four or more ANC visits. Further, health promotion may be more effective when CHWs get outcomes-linked monetary incentives as was the case with ASHA and in such cases, additional effect of counseling by other cadre of CHWs may not be realized. On the other hand, when there are no incentives for promoted health practices, coordination between different types of CHWs can be more effective. Future research could explore whether coordinated messaging where both CHWs get outcomes-linked incentives would be more effective.

Study implications

The outcomes of our study lend support for promotion of multisectoral collaboration in the form of coordinated counseling by leveraging the complementary roles of ASHAs and AWWs to advance maternal and child health. However, better clarity of roles and responsibilities associated with the CHWs could potentially lead to better service delivery, focusing on a specific set of outcomes [28]. Such clarity in roles may enable India to expedite progress toward achieving universal health coverage in line with the Sustainable Development Goals (SDG) targets.

Future research efforts stand to benefit greatly from the development of standardized definitions and measurement frameworks at a global level for concepts such as coordination, convergence, and collaboration among community-level functionaries and institutions. An example of this is the work being undertaken by Glandon and colleagues in the context of CHWs in India [50]. Finally, future research should examine the effectiveness of outcomes-linked incentives for AWWs and whether such incentives can complement the health promotion efforts led by ASHAs more effectively. Conversely, it is equally important to investigate whether nutrition-specific outcomes assigned to ASHAs can contribute to improved nutrition outcomes when coupled with counseling provided by AWWs.

ETHICS APPROVAL AND CONSENT TO PARTICIPATE

Study protocols were reviewed and approved by institutional review boards at the University of California, Berkeley (Ref. No. 2016-08-9092), and the India-based Suraksha Independent Ethics Committee (Protocol No. 2016-08-9092). The procedures for obtaining verbal audio-recorded informed consent from participants before data collection were approved by both the institutional review boards at the University of California, Berkeley and Suraksha Independent Ethics Committee. Verbal audio-recorded informed consent was obtained from the participants prior to data collection. All methods were carried out in accordance with the Declaration of Helsinki guidelines and regulations.

CONSENT FOR PUBLICATION.

Not applicable.

COMPETING INTERESTS

The authors declare that they have no competing interests.

DATA AVAILABILITY STATEMENT

The data that support the findings of this study are available on request from the corresponding author.

FUNDING

This study is funded by Grant No. OPP1158231 from Bill and Melinda Gates Foundation. Trial registration number: https://doi.org/10.1186/ISRCTN83902145

Registration date: 08/12/2016

AUTHORS' CONTRIBUTION

LG—conceptualization, formal analysis, writing (original draft), writing (reviewing and editing);
SRP— conceptualization, formal analysis, writing (reviewing and editing);
LCHF—writing (reviewing and editing) and funding;
DMW—writing (reviewing and editing) and funding;
ND-S—writing (reviewing and editing).
All authors read and approved the final manuscript.

ACKNOWLEDGMENTS

We extend our sincere thanks to the enumerators at NEERMAN for data collection. Most of all, we thank the survey respondents for their time. We also acknowledge contributions made by colleagues at International Food Policy and Research Institute, NEERMAN, University of California San Francisco, and University of California at Berkeley. We also thank Sneha Nimmagadda at NEERMAN for her efforts in cleaning and preparing the dataset.

References

1. Dandona R, Kumar GA, Henry NJ, Joshua V, Ramji S, Gupta SS, et al. Subnational mapping of under-5 and neonatal mortality trends in India: the Global Burden of Disease Study 2000–17. The Lancet. 2020;395:1640–58.

2. Meh C, Sharma A, Ram U, Fadel S, Correa N, Snelgrove J, et al. Trends in maternal mortality in India over two decades in nationally representative surveys. BJOG Int J Obstet Gynaecol. 2022;129:550–61.

3. Hug L, Alexander M, You D, Alkema L. National, regional, and global levels and trends in neonatal mortality between 1990 and 2017, with scenario-based projections to 2030: a systematic analysis. Lancet Glob Health. 2019;7:e710–20.

4. WHO, UNICEF, UNFPA, World Bank Group, United Nations Population Division. Trends in maternal mortality: 1990 to 2015. Geneva: WHO, UNICEF, UNFPA, World Bank Group, and United Nations Population Division; 2015.

5. Bhutta ZA, Das JK, Bahl R, Lawn JE, Salam RA, Paul VK, et al. Can available interventions end preventable deaths in mothers, newborn babies, and stillbirths, and at what cost? The Lancet. 2014;384:347–70.

6. Bhutta ZA, Das JK, Rizvi A, Gaffey MF, Walker N, Horton S, et al. Evidence-based interventions for improvement of maternal and child nutrition: what can be done and at what cost? Lancet Lond Engl. 2013;382:452–77.

7. Perry HB, Zulliger R, Rogers MM. Community Health Workers in Low-, Middle-, and High-Income Countries: An Overview of Their History, Recent Evolution, and Current Effectiveness. Annu Rev Public Health. 2014;35:399–421.

8. Kok MC, Dieleman M, Taegtmeyer M, Broerse JE, Kane SS, Ormel H, et al. Which intervention design factors influence performance of community health workers in low- and middle-income countries? A systematic review. Health Policy Plan. 2015;30:1207–27.

9. Haines A, Sanders D, Lehmann U, Rowe AK, Lawn JE, Jan S, et al. Achieving child survival goals: potential contribution of community health workers. The Lancet. 2007;369:2121–31.

10. Lassi ZS, Bhutta ZA. Community-based intervention packages for reducing maternal and neonatal morbidity and mortality and improving neonatal outcomes. Cochrane Database Syst Rev. 2015. https://doi.org/10.1002/14651858.CD007754.pub3.

11. World Health Organization. STANDARDS FOR IMPROVING QUALITY OF MATERNAL AND NEWBORN CARE IN HEALTH FACILITIES. World Health Organization; 2016.

12. Perry H, Zulliger R, Scott K, Javadi D, Gergen J. Case Studies of Large-Scale Community Health Worker Programs: Examples from Bangladesh, Brazil, Ethiopia, India, Iran, Nepal, and Pakistan. 2013.

13. Agarwal S, Curtis SL, Angeles G, Speizer IS, Singh K, Thomas JC. The impact of India's accredited social health activist (ASHA) program on the utilization of maternity services: a nationally representative longitudinal modelling study. Hum Resour Health. 2019;17:68.

14. Kosec K, Avula R, Holtemeyer B, Tyagi P, Hausladen S, Menon P. Predictors of Essential Health and Nutrition Service Delivery in Bihar, India: Results From Household and Frontline Worker Surveys. Glob Health Sci Pract. 2015;3:255–73.

15. Wang H, Juyal RK, Miner S, Fischer E. Performance-Based Payment System for ASHAs in India: What Does International Experience Tell Us? Bethesda, Maryland: Intrahealth International, Abt Associates; 2012.

16. John A, Nisbett N, Barnett I, Avula R, Menon P. Factors influencing the performance of community health workers: A qualitative study of Anganwadi Workers from Bihar, India. PLOS ONE. 2020;15:e0242460.

17. Gopalakrishnan L, Diamond-Smith N, Avula R, Menon P, Fernald L, Walker D, et al. Association between supportive supervision and performance of community health workers in India: a longitudinal multi-level analysis. Hum Resour Health. 2021;19:145.

18. Ministry of Health & Family Welfare, Government of India. ASHA Workers Incentive Structure, Ministry of Health and Family Welfare. 2020. https://pib.gov.in/Pressreleaseshare.aspx?PRID=1606212. Accessed 10 Sep 2023.

19. Smittenaar P, Ramesh BM, Jain M, Blanchard J, Kemp H, Engl E, et al. Bringing Greater Precision to Interactions Between Community Health Workers and Households to Improve Maternal and Newborn Health Outcomes in India. Glob Health Sci Pract. 2020;8:358–71.

20. Wagner A, Bettampadi D, Porth J, Boulton M. The impact of increased use of ASHAs on rural immunization coverage in India. Int J Infect Dis. 2016;45:209–10.

21. Wagner AL, Porth JM, Bettampadi D, Boulton ML. Have community health workers increased the delivery of maternal and child healthcare in India? J Public Health Oxf Engl. 2018;40:e164–70.

22. Scott K, Beckham SW, Gross M, Pariyo G, Rao KD, Cometto G, et al. What do we know about community-based health worker programs? A systematic review of existing reviews on community health workers. Hum Resour Health. 2018;16:39.

23. Nadella P, Subramanian SV, Roman-Urrestarazu A. The impact of community health workers on antenatal and infant health in India: A cross-sectional study. SSM - Popul Health. 2021;15:100872.

24. Chakrabarti S, Raghunathan K, Alderman H, Menon P, Nguyen P. India's Integrated Child Development Services programme; equity and extent of coverage in 2006 and 2016. Bull World Health Organ. 2019;97:270–82.

25. International Institute for Population Sciences (IIPS) and ICF. National Family Health Survey (NFHS-5), 2019-21. Demographic and Health Survey. Mumbai: International Institute for Population Sciences; 2021.

26. Rammohan A, Goli S, Saroj SK, Jaleel CPA. Does engagement with frontline health workers improve maternal and child healthcare utilisation and outcomes in India? Hum Resour Health. 2021;19:45.

27. Burger O, Hashmi F, Dańko MJ, Akhauri S, Chaudhuri I, Little E, et al. Facilitating behavioral change: A comparative assessment of ASHA efficacy in rural Bihar. PLOS Glob Public Health. 2022;2:e0000756.

28. Kim SS, Avula R, Ved R, Kohli N, Singh K, van den Bold M, et al. Understanding the role of intersectoral convergence in the delivery of essential maternal and child nutrition interventions in Odisha, India: a qualitative study. BMC Public Health. 2017;17:161.

29. Jamison DT, Alwan A, Mock CN, Nugent R, Watkins DA, Adeyi O, et al. Universal Health Coverage and Intersectoral Action for Health. In: Jamison DT, Gelband H, Horton S, Jha P, Laxminarayan R, Mock CN, et al., editors. Disease Control Priorities: Improving Health and Reducing Poverty. 3rd edition. Washington (DC): The International Bank for Reconstruction and Development / The World Bank; 2017.

30. Rasanathan K, Damji N, Atsbeha T, Drisse M-NB, Davis A, Dora C, et al. Ensuring multisectoral action on the determinants of reproductive, maternal, newborn, child, and adolescent health in the post-2015 era. BMJ. 2015;351:h4213.

31. Hinton R, Armstrong C, Asri E, Baesel K, Barnett S, Blauvelt C, et al. Specific considerations for research on the effectiveness of multisectoral collaboration: methods and lessons from 12 country case studies. Glob Health. 2021;17:18.

32. National Health Mission, GoI. Intersectoral convergence between DWCD and DHFW - DOHFW :: National Health Mission. https://www.nhm.gov.in/index1.php?lang=1&level=2&sublinkid=1084&lid=149. Accessed 11 Aug 2023.

33. Nimmagadda S, Gopalakrishnan L, Avula R, Dhar D, Diamond-Smith N, Fernald L, et al. Effects of an mHealth intervention for community health workers on maternal and child nutrition and health service delivery in India: protocol for a quasi-experimental mixed-methods evaluation. BMJ Open. 2019;9:e025774.

34. Patil SR, Nimmagadda S, Gopalakrishnan L, Avula R, Bajaj S, Diamond-Smith N, et al. Can digitally enabling community health and nutrition workers improve services delivery to pregnant women and mothers of infants? Quasi-experimental evidence from a national-scale nutrition programme in India. BMJ Glob Health. 2022;6 Suppl 5:e007298.

35. Williams R. Using the Margins Command to Estimate and Interpret Adjusted Predictions and Marginal Effects. Stata J. 2012;12:308–31.

36. Dunn OJ. Multiple Comparisons among Means. J Am Stat Assoc. 1961;56:52–64.

37. StataCorp,. Stata Statistical Software: Release 15. 2017.

38. MoHFW G of I. Guidelines on Accredited Social Health Activists (ASHA). Ministry of Health and Family Welfare.

39. MWCD, Government of India. Roles and responsibilities of AWWs. Ministry of Woman and Child Development.

40. Scott K, George AS, Ved RR. Taking stock of 10 years of published research on the ASHA programme: examining India's national community health worker programme from a health systems perspective. Health Res Policy Syst. 2019;17:29.

41. Akshaya KM, Shivalli S. Birth preparedness and complication readiness among the women beneficiaries of selected rural primary health centers of Dakshina Kannada district, Karnataka, India. PLOS ONE. 2017;12:e0183739.

42. Olaniran A, Madaj B, Bar-Zev S, van den Broek N. The roles of community health workers who provide maternal and newborn health services: case studies from Africa and Asia. BMJ Glob Health. 2019;4:e001388.

43. Agarwal S, Curtis S, Angeles G, Speizer I, Singh K, Thomas J. Are community health workers effective in retaining women in the maternity care continuum? Evidence from India. BMJ Glob Health. 2019;4:e001557.

44. Jain M, Caplan Y, Ramesh BM, Kemp H, Hammer B, Isac S, et al. Improving Community Health Worker Compensation: A Case Study From India Using Quantitative Projection Modeling and Incentive Design Principles. Glob Health Sci Pract. 2022;10:e2100413.

45. Sharma A, Khapre M, Meshram R, Gupta A. Determinants of utilization of antenatal care services among recently delivered women residing in urban poor areas of Rishikesh, Uttarakhand, India—a cross-sectional study. J Rural Med JRM. 2023;18:87–95.

46. Kumar G, Choudhary TS, Srivastava A, Upadhyay RP, Taneja S, Bahl R, et al. Utilisation, equity and determinants of full antenatal care in India: analysis from the National Family Health Survey 4. BMC Pregnancy Childbirth. 2019;19:327.

47. Singh L, Dubey R, Singh PK, Nair S, Rai RK, Vardhana Rao MV, et al. Coverage of Quality Maternal and Newborn Healthcare Services in India: Examining Dropouts, Disparity and Determinants. Ann Glob Health. 2022;88:39.

48. Lincetto O, Mothebesoane-Anoh S, Gomez P. Opportunities for Africa's Newborns: Practical data, policy and programmatic support for newborn care in Africa. World Health Organization; 2006.

49. Carmichael SL, Mehta K, Raheel H, Srikantiah S, Chaudhuri I, Trehan S, et al. Effects of team-based goals and non-monetary incentives on front-line health worker performance and maternal health behaviours: a cluster randomised controlled trial in Bihar, India. BMJ Glob Health. 2019;4:e001146.

50. Glandon D, Leoutsakos J-M, Gupta S, Marsteller J, Paina L, Bennett S. Development and psychometric testing of the FLW-MSC scale for measuring frontline worker multisectoral collaboration in rural India. BMJ Open. 2021;11:e037800.

Click here to access/download
Supporting Information
Supplementary Material.docx
