# Supplementary material for: Association between coordinated counseling from both ASHA and Anganwadi Workers and maternal health outcomes: A cross-sectional study from Madhya Pradesh and Bihar, India
Source: PLOS Glob Public Health. 2024 Nov 13;4(11):e0003639. doi: 10.1371/journal.pgph.0003639 (PMC11560015; doi:10.1371/journal.pgph.0003639)
Supplement: Attachment — Submitted filename: Response_to_comments.docx [file pgph.0003639.s004.docx]

**Response to Reviewers**

Dear reviewers,

Thank you so much for your keen feedback on improving our manuscript. Please see the reply to each reviewer below in blue colored font. Specific text added to the manuscript is in *italics.*

**Reviewer #1:**

Comments:

1. As study is done in 2 state referring to entire India might be not appropriate

Edited to “Association between coordinated counseling from both ASHA and Anganwadi Workers and maternal health outcomes in : A cross-sectional study from Madhya Pradesh and Bihar, India.”

2. Only ANC and PNC were covered. evidence suggested care during delivery, mother nutrition, family support, social support etc play a major role in 1000 days outcome.

Thank you, we added more citations to suggest the importance of care during delivery, maternal nutrition and social support in improving health and well-being outcomes in the first 1000 days of life.

3. Clarify which are the two flagship national programs.

Clarified. Added a line -- *India has two flagship national programs, including the National Health Mission (NHM) and Integrated Child Development Services (ICDS) with combined workforce of more than 2 million female CHWs providing maternal health and nutrition promotion and allied services.*

4. CHW role is differ from state to state. As health and social welfare is a state subject. Request to re look the roles in Bihar and MP. In India, health is a state subject. It would be beneficial to provide a brief overview of the health systems in Bihar and Madhya Pradesh.

While we understand health is a state subject, the NHM guidelines are followed in both in Bihar and MP. Instead we provide the context of the indicators in MP and Bihar:

*Both Madhya Pradesh (MP) and Bihar face significant health challenges. Under-five mortality rates are high, with 69 per 1000 live births in MP and 60 per 1000 live births in Bihar. Stunting affects 44% of children under five in MP and 49% in Bihar. Anemia prevalence is 55% among pregnant women and children in both states. Educational attainment for women aged 15-49 is low, with only 14% in MP and 12% in Bihar completing 12 or more years of schooling [40]. Antenatal and delivery-related indicators are poorer in Bihar compared to MP. For instance, 36% of mothers in MP had at least four ANC visits, compared to only 14% in Bihar. Institutional deliveries were more common in MP (81%) than in Bihar (64%). However, Bihar outperformed MP in child immunization, with 62% of children aged 12-23 months fully immunized in Bihar compared to 50% in MP [40]. The differences in antenatal care visits and institutional deliveries suggest that MP has somewhat better maternal health service uptake compared to Bihar. This could reflect differences in healthcare access, quality, or health-seeking behaviors between the two states.*

5. Data was collected before 2019 not sure about covid-19 relevance in introduction referring to this line “There is growing recognition that the current challenges and inequities experienced by women and children will likely continue to be worsened by the impact of COVID-19, necessitating the imperative for multisectoral collaboration.”

Thank you for your observation regarding the COVID-19 reference in our introduction. You are correct that our data collection predates the pandemic. The mention of COVID-19 was included not to directly relate to our data, but to contextualize the ongoing importance of our research findings in the current and future public health landscape. This line is merely emphasizing the need for more collaborative care and multisectoral collaboration.

6. Before explaining hypothesis, its better to explain public health care system. Author completely undermine the role of health care workers in pregnant women care.

We appreciate the reviewer's concern about explaining the public health care system and the role of healthcare workers in pregnant women's care. However, we respectfully disagree that this aspect has been undermined or requires additional elaboration in our manuscript for the following reasons:

Our research specifically focuses on community or frontline health workers and their (impact) role in providing care (counseling in our case) to pregnant and lactating women. This focus is clearly stated, and the introduction already provides a comprehensive overview of the relevant aspects of the public health care system as they pertain to our study's focus. We have detailed the roles of ASHAs and AWWs, who are key frontline health workers in the Indian context. While we acknowledge the broader healthcare system's importance, an extensive explanation of the entire public health care system would extend beyond the scope of our study and potentially detract from our primary research questions.

7. Need to elaborate. Why oral consent? Did the witness presented during oral consent process? Did the participants receive information sheet?

Thank you for your questions regarding our consent process. We opted for oral (audio-recorded) informed consent, which was approved by IRBs both in the US and India. This choice was made for several important reasons:

- Literacy considerations: Many of our study participants were semi-literate, making verbal consent more appropriate and inclusive.
- Cultural sensitivity: In India, there's often a cultural hesitancy or fear associated with signing documents, especially for surveys or interviews. Verbal consent helps alleviate these concerns and makes participants more comfortable.
- Procedural clarity: After verbally agreeing to proceed with the interview, participants were asked to read or repeat the following sentence: "I have understood the purpose of the interview and my rights as a respondent. I agree to participate in this interview." This ensured clear understanding and agreement.

We did not require a witness during the consent process because the consent was audio-recorded by our enumerators, providing a verifiable record of each participant's agreement. The audio recordings of consent were stored securely in compliance with data protection regulations. Before administering the survey, all participants received an information and consent sheet, ensuring they had access to full details about the study. This approach to consent is commonly used in similar research contexts in India, balancing ethical requirements with cultural sensitivity and practical considerations. We have published several manuscripts from this study, and this did not come up as an objection in any of the journals.

We added an abbreviated answer to the manuscript.

8. Add citation for “principal components analysis”

We have added the citation for PCA.

9. It will good to name the variable names, instead of general explanation -- “We describe sample characteristics in terms of frequencies, proportions and/or median and interquartile range (IQR) of the exposure and outcome indicators and covariates discussed previously. For each outcome, we fit a fixed-effects logistic regression adjusted for covariates which could potentially bias the estimated association between the outcome and counseling from CHWs. Since the mHealth intervention was assigned at the district level, the fixed effect at sub- district level ensures that the associations are estimated in the original treatment and controlled sub-districts separately and then averaged over.”

We listed the outcomes but did not do the same for exposure and covariates since it was covered just before this section.

10. Clarify Stata version.

Stata V 15.0; added in the paper as well.

11. Authors completely ignored Health care workers role in Mother care and in decision making process both in analysis or in limitations.

While our study did not explicitly focus on this aspect, we recognize its significance and agree that it deserves attention. To address your concern, we added the following statement in the limitations section of our paper acknowledging that our study did not directly examine the role of healthcare workers in maternal care and decision-making processes. This will help readers understand the scope of our analysis and potential areas for future research.

*“We acknowledge that our study's scope did not extend to directly examining the role of healthcare workers in the decision-making processes of mothers regarding their care-seeking behaviors. This important aspect of maternal healthcare deserves dedicated attention in future research to provide a more comprehensive understanding of the factors influencing maternal health outcomes.”*

12. The use of fixed effects in the analysis was unclear. Consider conducting a stratified analysis comparing intervention and non-intervention areas of mHealth.

Thank you for your thoughtful suggestion. Indeed, separate sub-group analysis is sometimes done and can be helpful. However, we respectfully submit that, our present modelling approach is most appropriate and robust. The sampling was not stratified by treatment and control group but a propensity 1:1 score matched sample that identified pairs of villages to control for all village- and above-village time-invariant confounders – whether observed or not. Breaking this pair and separating treatment and control groups would substantially weaken this strength. Two sub-group level analysis would also mean that we have assumed independence of these two sub-groups which is not the case due to our sampling approach.

**Reviewer 2 comments**

1. Please consider introducing and describing the ANM earlier in the manuscript. The ANM should especially be discussed when you mention government efforts to encourage collaboration between ASHAs and AWWs. The official policy that promotes cooperation between the ASHA and AWW also involves the ANM (the “triple A” collaboration), so mentioning the ANM there makes a lot of sense. Consider also explaining earlier in the paper why you did not consider the additive role of the ANM as well.

Thank you. We introduced the ANM and triple-A concepts early on. In the methods section, we specifically explained why ANM was not added to the models.

We added why ANM was not included in the methods section – “The exclusion of Auxiliary Nurse Midwives (ANMs) from our analysis was a deliberate methodological decision based on the fundamental differences in their role and operational scope compared to ASHAs and AWWs. ANMs primarily focus on clinical service provision, including health check-ups, referrals, and immunizations, and are responsible for a significantly larger population (over 10,000) spread across 4-5 villages. This broader scope inherently limits their capacity for intensive, village-level health promotion activities [14]. In contrast, ASHAs and AWWs operate exclusively at the village level, allowing them to provide more focused, consistent, and personalized health promotion and counseling services. Their community-embedded nature enables them to engage in frequent, direct interactions with individual families, which is central to the health promotion activities examined in our study.”

2. Could you provide insight into how survey enumerators moved from women’s natural language responses (e.g., “ASHA talked to me after I had my baby”) to specific coded response categories (i.e., was this about postnatal care or postpartum family planning?”).

The survey enumerators were trained to specify the life stage related to which the questions were being asked. Additionally, the survey questions specifically asked the women -- *After the delivery of [**INDEXCHILD**], did you receive the following services? For example, advice on family planning(postpartum family planning) or advice on health check-up (postnatal care) From whom?*

3. Please re-read for typos throughout. The “overlapping responsibilities” row is presented twice. “Contrary to our hypothesis, coordinated counseling on counseling on health issues…” Sometimes you write “percent”, sometimes “pp”, sometimes “%”.

Apologies for this oversight. We have thoroughly re-read the entire manuscript and corrected all identified typos. The redundant "overlapping responsibilities" row has been removed. We have standardized our use of percentage notation throughout the manuscript. We now consistently use “%” for percentages and “pp” for percentage points. Thank you for noting this distinction; percentage (%) and percentage point (pp) are different metrics, and we have ensured they are used appropriately throughout.

4. Under “payment structure” could you list all the ASHA payments for the health-related behaviors that you focus on in this paper?

We have updated Table 1 to include the payment structure for ASHA workers related to the health behaviors we focus on in this paper. Regarding birth preparedness, we conducted a thorough review of the available documentation on ASHA incentives but could not find a specific payment linked to this activity. It appears that birth preparedness counseling may be considered part of the overall antenatal care package rather than a separately incentivized task.

5. In the methods section, could you clarify what behavior women had to report to be considered to have practiced birth preparedness? The other variables are clear to me (4+ ANC, ID, PNC) but birth preparedness seems less black-and-white.

Added this explanation to all the variables to keep it consistent --- “Birth preparedness was coded 1 if at least 75% of birth preparatory practices were followed by the woman /mother prior to the birth of indexchild, including identification of health facility for the delivery or in case of emergency during delivery, obtaining clean cloth for drying the baby, obtaining new blade to cut cord, saving money for delivery, and arranging transport.”

6. Regarding “Contrary to our hypothesis, coordinated counseling on counseling on health issues/danger signs during pregnancy is not statistically significantly associated (-1.3 pp [95%CI: -4.2 – 1.4 pp]) with mothers receiving four or more ANC check-ups compared to the reference group mean of 36.8 percent. Standalone counseling from only ASHA or only AWW are also not associated with mothers receiving four or more ANC.” ◊ Depending on the exact nature of the “counseling on health issues and danger signs,” ASHAs and AWWs may have presented information to encourage pregnant women to have institutional deliveries rather than to encourage 4+ ANC. This could explain why counseling on danger signs was not associated with ANC uptake.

Thank you for this insightful observation. We agree that the nature of counseling provided by ASHAs and AWWs could indeed explain the lack of association between counseling on check-up and ANC uptake. Your suggestion that they may prioritize topics like institutional delivery over four or more ANC visits is a valuable perspective we hadn't previously considered.

*“The lack of association between counseling on health issues during pregnancy and mothers receiving four or more ANC check-ups may be explained by the prioritization of counseling topics. ASHAs and AWWs might focus more on encouraging institutional deliveries, which they may perceive as more critical for maternal health outcomes, rather than emphasizing the importance of four or more ANC visits. This potential prioritization could be influenced by various factors, including perceived urgency of different health behaviors, time constraints during counseling sessions, or even the structure of incentives for health workers.”*

7. In regards to “A national-level analysis of the ASHA program had found that while ASHAs were effective in helping women initiate their maternity care through any ANC, ASHAs were less effective in ensuring completion of services along the continuum of care such as four or more ANC visits.” ◊ There is also the issue of whether the 4+ ANC visits were available for women or not. I.e., whether the ANM come to the village for at least four VHNDs during the woman’s pregnancy. Would it make sense to note here that not everything depends on the woman wanting it or the ASHA counseling on it – it also depends on the health system providing it. You rightly mention low quality or negative experiences with ANC, but maybe it is worth considering availability too.

Thank you for this crucial observation, this is such an important point. Added the following to the discussion section.

*“Furthermore, the uptake of 4+ anternatal care (ANC) visits is not solely dependent on women's desire for care or ASHA's counseling effectiveness. It is also contingent on the health system's capacity to provide these services. The consistent presence of Auxiliary Nurse Midwives (ANMs) in villages for at least four Village Health and Nutrition Days during a woman's pregnancy is crucial. This highlights that while demand-side factors are important, supply-side constraints within the health system play a pivotal role in determining ANC utilization.”*

8. Regarding: “Future research could explore whether coordinated messaging where both CHWs get outcomes-linked incentives would be more effective.” And “Finally, future research should examine the effectiveness of outcomes-linked incentives for AWWs and whether such incentives can complement the health promotion efforts led by ASHAs more effectively.” ◊ I agree with you. Financial incentives have immediate impact on behavior, particularly for marginalized women who work for very low pay. However I want to suggest up an alternative mentality around counseling and CHW performance, that draws from a framework of intrinsic motivation, worker’s rights, and trust. Might we also consider that when CHWs counsel women to perform behaviors not linked to incentives, the CHW may have a stronger intrinsic belief in this behavior’s importance or the woman may place greater weight on this message (because they know that the CHW isn’t pushing for compensation)? Do we want to break everything down into incentives in order to get CHWs to perform? I am not sure, but I wanted to mention this alternative, and perhaps more critical, perspective for your consideration. You may find this article by Ved and Scott useful (<https://www.ghspjournal.org/content/8/3/332.abstract>) and may want to bring in a bit more literature on CHW rights and incentives.

Thank you for this insightful and thought-provoking comment. You've raised a crucial point about the complex nature of motivation in community health work, which indeed deserves more attention. This is something I have personally heard about in my previous qualitative experience with CHWs and beneficiaries and resonates strongly with the real-world dynamics I've observed in the field, where the relationship between CHWs and community members often goes beyond simple service provision. I enjoyed reading the paper you suggested and have added this point about the importance of intrinsic motivation and trust between CHWs/ASHAs and beneficiaries to our discussion. We've also added the reference you suggested: Ved, R., & Scott, K. (2020). Advancing the science and practice of community health worker performance measurement. Global Health: Science and Practice, 8(3), 332-334.

I added the following to the paper:

*“This also raises important questions about the role of intrinsic motivation and trust in CHW-community relationships. When CHWs counsel on behaviors not linked to incentives, it may reflect a stronger intrinsic belief in the behavior’s importance and potentially carry more weight with women, as it is not driven by compensation motives. While financial incentives play a role in shaping ASHA or CHW behavior, factors such as intrinsic motivation, community trust, and health system functionality are equally crucial in determining the program's success in improving community health outcomes [57].*

*Future research could also explore whether coordinated messaging where both CHWs get outcomes-linked incentives would be more effective, while also considering the potential benefits of non-incentivized counseling in building trust and promoting intrinsic motivation among both CHWs and community members.”*
